# Utilization of Robust Zr-Based Metal–Organic Framework for Efficient N_2_/H_2_ Separation

**DOI:** 10.3390/ma19112418

**Published:** 2026-06-05

**Authors:** Jieru Zhang, Xia Chen, Zhilu Wang, Tianhao Wang, Wenxin Ma, Qiang Fu, Bao-Ju Wang

**Affiliations:** School of Chemistry and Chemical Engineering, Shandong University of Technology, Zibo 255000, China; zhangjieru98@163.com (J.Z.); sevenwangzl@163.com (Z.W.); w1548942696@163.com (T.W.); mwxo3o@163.com (W.M.)

**Keywords:** adsorptive separation, nitrogen, hydrogen, molecular dynamic simulation, UiO MOFs

## Abstract

The development of energy-efficient separation technologies for nitrogen (N_2_)–hydrogen (H_2_) mixtures under conventional industrial conditions remains a critical challenge. Adsorptive separation emerges as a promising strategy, contingent upon the design of high-performance adsorbents with tailored physicochemical properties. In this study, three isoreticular zirconium-based metal–organic frameworks (UiO-66, UiO-66-CH=CH_2_, and UiO-66-F_4_) are systematically evaluated for the purification of industrial N_2_-H_2_ mixed gases. By integrating static adsorption isotherms, dynamic breakthrough experiments, and molecular dynamics (MD) simulations, we comprehensively characterize the adsorption mechanisms and diffusion kinetics. The results demonstrate that UiO-66-F_4_ exhibits optimal performance in capturing N_2_ from N_2_-H_2_ mixtures under simulated industrial conditions, owing to its balanced binding affinity, high nitrogen uptake, and favorable micro-/mesoporous diffusion dynamics. This work offers a promising alternative for N_2_-H_2_ separation in industrial applications.

## 1. Introduction

As a kind of essential fuel and energy carrier, hydrogen has been widely applicated in modern industry and emerging energy sources due to having the advantage of zero emissions and being pollution-free [1,2,3,4,5]. Industrial sources of hydrogen include coal gasification, methane reformation, water electrolysis and industrial by-products such as biomass, chlor-alkali, and synthetic ammonia [6,7,8,9,10]. For example, recovering hydrogen from ammonia purge gas is an economic and environmentally benign method [10]. However, the nonpolar N_2_/H_2_ pair is one of the most difficult to be separated due to their similar molecular sizes (N_2_: 3.64 Å, H_2_: 2.89 Å), similar quadrupole moments (N_2_: 4.7 × 10^−40^ C·m^2^, H_2_: 0 C·m^2^), and similar physicochemical properties [8,11]. Compared with other feasible gas separation and purification methods such as condensation, cryogenic rectification, and membrane separation, the technology of adsorptive separation possesses advantages of high efficiency and low consumption, simple technology, and lower industrial-scale construction costs considering the present technical state [12,13,14,15]. Adsorbents with excellent adsorption properties are crucial to the adsorptive separation technique [7,16,17]. As a result, the development of novel adsorptive separation materials, which are more energy efficient than existing materials, will have a significant impact on the economic and environmental costs associated with global H_2_ production.

Metal–organic frameworks (MOFs) have become a class of representative new porous materials in the fields of molecular capture/adsorptive separation due to their characteristics of flexible and tunable structure, functionally modified pore space, high specific area, and permanent high porosity [18,19,20,21,22,23,24]. Despite extensive research on MOFs, the vast majority focus on synthesis, with limited reports addressing their performance for hydrogen purification, particularly the separation of N_2_-H_2_ mixtures, and even fewer investigating this process under dynamic flow conditions [25,26,27,28,29]. Furthermore, the substantial adsorbent quantities required for industrial applications necessitate frameworks that combine excellent adsorption properties with straightforward synthesis to offset operational costs. Zirconium-based MOFs represented by UiO-66 (UiO for University of Oslo) are a fascinating family of robust porous materials with excellent stability, rapid adsorption, low production cost, and ease of regeneration [12,30,31,32]. This makes these frameworks highly attractive for commercial applications. Accordingly, we selected several isostructural UiO-66-type MOFs as candidates to evaluate their performance in separating N_2_-H_2_ mixtures. Previous research suggests that the affinity between the frameworks and H_2_ molecules can be enhanced by exposing a high concentration of open metal sites (OMS) and expanding the π-conjugated system formed by ligand [25,28,33]. Complementing this, our prior work demonstrates that the affinity between the frameworks and N_2_ molecule was enhanced by introducing F atoms with strong electron-withdrawing ability to the F-pillared-layer frameworks [8]. This enhancement arises because N_2_ molecules possess a denser electron cloud distribution compared to H_2_ molecules, leading to stronger induction interactions between N_2_ molecules and F atoms of the framework [8,34]. Building upon these insights, three analogs with interpenetrative structure, namely UiO-66, UiO-66-CH=CH_2,_ and UiO-66-F4 were selected for adsorptive separating N_2_ from H_2_ molecules.

Herein, the adsorptive separation of N_2_-H_2_ mixture over Zr-based MOFs (UiO-66, UiO-66-CH=CH_2,_ and UiO-66-F_4_) was investigated from a thermodynamics and kinetics perspective by combining adsorption experiments and molecular dynamic (MD) simulation. The performance of these hierarchically porous materials was characterized through pure component N_2_/H_2_ adsorption isotherms and high-throughput dynamic breakthrough experiments with binary mixtures. Experimental and simulation results jointly demonstrate that UiO-66-F_4_ exhibits the longest breakthrough time and moderate N_2_ adsorption capacity, rendering it highly suitable for N_2_/H_2_ separation. Its superior separation performance stems from the combined effects of binding site affinity, guest molecule adsorption capacity, and diffusion kinetics within its micro-mesoporous structure. Furthermore, UiO-66-F_4_’s excellent cyclic stability and recyclability underscore its potential for industrial hydrogen purification from N_2_-H_2_ mixtures.

## 2. Experimental Section

### 2.1. Materials and Characterization

All raw materials and reagents used for synthesis were purchased from commercial sources and used without further purification. The used gases had the following ultra-high purity: H_2_ (>99.999%), N_2_ (>99.999%), and He (>99.999%) was purchased from BaiYan Co., Ltd. (Zibo, China). Powder X-ray diffraction (PXRD) data were collected at room temperature by using a conventional high-resolution (θ–2θ) diffractometer (D8-ADVANCED, Bruker, Germany) with Cu-Kα radiation at a scanning rate of 0.2° s^−1^ from 3° to 50°. The relevant pore characteristics of the adsorbents (BET specific surface area, pore volume, pore size) are obtained from N_2_ adsorption at 77 K and relative pressure (P/P_0_) up to 1 using a Micromeritics 3Flex surface characterization analyzer (Micromeritics Instrument Corporation, Norcross, GA, USA). FT-IR spectra of the samples were recorded by a Nicolet 5700 spectrometer (Thermoelectric, Waltham, MA, USA) in the range of 50–7800 cm^−1^.

### 2.2. Synthesis of UiO-66 Type Zirconium-Based MOFs

**Scheme 1:**
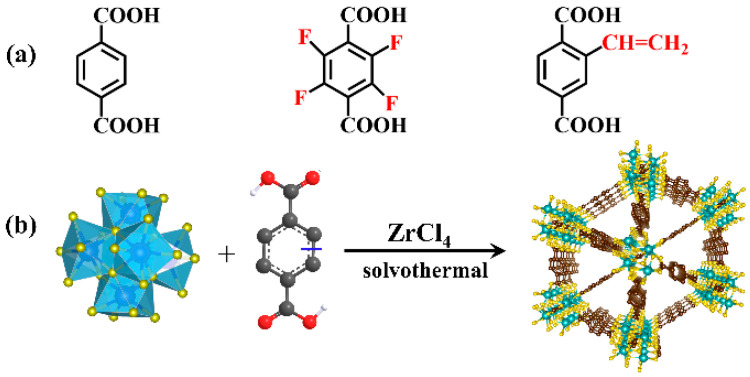
(**a**) Chemical structures of the organic linkers used in this work (from left to right: terephthalic acid, 2-vinylterephthalic acid, and 2,3,5,6-tetrafluoroterephthalic acid); (**b**) Synthesis of Zr-based MOFs.

Isostructural UiO-66 samples were synthesized via a modified solvothermal protocol based on established methodologies [30,35,36,37,38], utilizing ZrCl_4_ as the metal precursor (Scheme 1). Specifically, HBr acid-mediated modulation was incorporated during synthesis, resulting in UiO-66-nHBr variants (where n denotes the molar ratio of HBr to ZrCl_4_). Two isostructural MOF derivatives with distinct pore architectures were obtained: UiO-66-1HBr (U_1_) and UiO-66-5HBr (U_5_). Additionally, UiO-66-CH=CH_2_ (Uvinyl) and UiO-66-F_4_ (UF_4_) were prepared by introducing 2-vinylterephthalic acid and 2,3,5,6-tetrafluoroterephthalic acid ligands, respectively. All as-synthesized materials underwent post-synthetic activation via vacuum treatment at 423 K for 12 h prior to characterization and adsorption performance evaluation.

### 2.3. Pure-Component Adsorption Experiments

**Figure 1:**
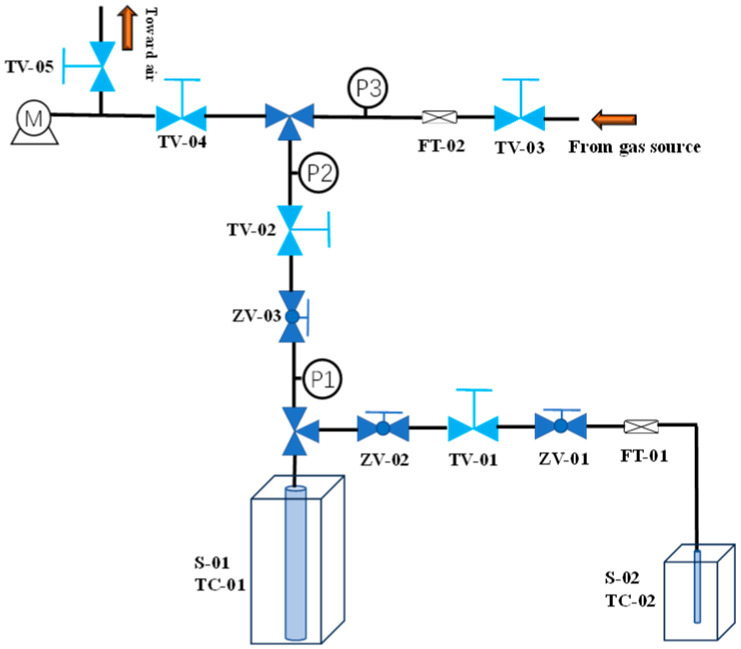
Schematic representation of the static volumetric experimental set-up. P1, P2, P3: Digital pressure transducers (KELLER LE02 PSIA). M: Dry diaphragm vacuum pump (ULVAC DAP-6D). FT-01, FT-02: Filter (SWAGELOK SS-SCF3-VR4-P-225). TV-01, TV-02, TV-03, TV-04: Needle valve (HANLOK). TV-05: Leak valve (HANLOK). ZV-01, ZV-02, ZV-03: Globe valve (HANLOK). S-01, S-02: Reference slot (10 mL) and adsorption slot (1 mL), respectively (SHANGHAI HUOTNG). TC-01, TC-02: Low temperature thermostatic circulator (HAISHU YIHENG, YHX-08).

Pure-component adsorption measurements were performed at pressures spanning from 0 to 4000 kPa using a self-assembled static volumetric apparatus (equipped with key components from Shanghai Huotong Experimental Instrument Co., Ltd., Shanghai, China), as illustrated in Figure 1. Approximately 500 mg of sample was loaded into the adsorption slot, after which the entire system was hermetically sealed. Prior to the determination of adsorption isotherms, the free-space volume was measured utilizing helium at room temperature [39]. The gas uptake was calculated based on the material balance principle when expanding a known amount of gas from the reference slot to the adsorption slot. The criterion for achieving adsorption equilibrium was defined as a pressure change of less than 0.5% within 600 s. Subsequently, the gas adsorption amounts on the sample at various pressures were calculated [40,41]. Before each test, the entire sealed system was evacuated for 30 min.

### 2.4. Isotherm Fitting

Based on the calculation-derived characteristics of experimental data, optimized fitting parameters were systematically determined for curve fitting. This study employed the Langmuir (Equation (1)) [42] and Freundlich (Equation (2)) [43] models to fit the single-component adsorption isotherms of N_2_ and H_2_, respectively. Through comparative analysis of the plotted adsorption isotherms and fitting curves, the influence patterns of modified materials on the adsorption performance of H_2_ and N_2_ were systematically elucidated.

(1) qeq=qmpp+1b

(2) $$q_{eq}=kp^{t}$$

where *q_eq_* is the gas uptake (mmol/g), *q_m_* is Langmuir volume reflecting the maximum uptake of the adsorbent (mmol/g), *p* is pressure (kPa), *b* (kPa^−1^) and *k* (mmol g^−1^ kPa^−t^) are constants associated with temperature and adsorption heat, and *t* is a constant related to temperature, pore distribution, and adsorption intensity.

### 2.5. Isosteric Heat (Qst) and Ideal Adsorption Selectivity (SN_2_/H_2_)

To determine the coverage-dependent isosteric heat of adsorption (Q_st_), the pure-component adsorption isothermal data were modeled with the Virial expansion method [28,44]. The adsorption equilibrium was described by the following equation:

(3) $$llnP=lnN+\frac{1}{T}\sum_{i=0}^{m}a_{i}N^{i}+\sum_{j=0}^{n}b_{j}N^{j}$$

where *P* (kPa) represents the equilibrium pressure, *N* (mg/g) denotes the gas uptake, *T* (K) is the temperature, and *a_i_* and *b_i_* are temperature-independent virial parameters [44,45]. The parameters m and n were optimized to ensure a robust fit to the experimental isotherm data. Subsequently, the Q_st_ was calculated via the following equation:

(4) $$Q_{st}=-R\times \sum_{i=0}^{m}a_{i}N^{i}$$

where *R* is the gas constant (8.314 J mol^−1^ K^−1^). This approach enables the extraction of energy landscapes governing adsorbate–adsorbent interactions under varying coverage conditions.

To predict competitive adsorption behavior in multicomponent systems, the Ideal Adsorbed Solution Theory (IAST) was employed to evaluate the adsorption separation selectivity of N_2_-H_2_ mixture (S_N2_/_H2_) on Zr-based MOFs. The IAST model integrates the pure-component adsorption isotherms derived from the Langmuir and Freundlich models, with fitting parameters obtained from the virial expansion analysis. The selectivity was quantified using the following expression:

(5) $$S_{N_{2}/H_{2}}=\frac{x_{N_{2}}/x_{H_{2}}}{y_{N_{2}}/y_{H_{2}}}=\frac{q_{N_{2}}/q_{H_{2}}}{p_{N_{2}}/p_{H_{2}}}$$

where *q_i_* and *p_i_* are the molar loading and partial pressure of component *i* in equilibrium, respectively. This framework provides a theoretical basis for evaluating the separation efficiency of Zr-MOFs under mixed-gas conditions, bridging single-component adsorption data to practical gas separation scenarios.

### 2.6. Dynamic Column Breakthrough Experiments

Dynamic column breakthrough experiments were conducted on a commercial multicomponent gas adsorption system (Mixsorb S, 3P INSTRUMENT, Germany) integrated with a mass spectrometer (RGA, MKS, USA) as the detection unit. The cylindrical adsorption column featured an inner diameter of 4.57 mm and an effective length of 45 mm. Prior to experimentation, the adsorbent underwent thermal activation under He flowing (10 mL·min^−1^) at 473 K (external heater setting, corresponding to column temperature ≈ 413 K) for 6 h. Subsequent breakthrough tests were performed under three distinct feed conditions: (1) H_2_/N_2_/He (10/10/80, vol%; carrier gas: He) at 30 mL·min^−1^ total flow rate; (2) H_2_/N_2_/He (10/20/70, vol%) at 30 mL·min^−1^; (3) H_2_/N_2_/He (20/10/70, vol%) at 25 mL·min^−1^. All experiments were executed under controlled pressure and temperature regimes. Post-test regeneration of the adsorbent was achieved through He purge (10 mL·min^−1^) at 453 K (external heater setting, column temperature ≈ 393 K) for 3 h prior to each successive run. The experimental sequence ensured complete desorption of previously adsorbed species between cycles, maintaining adsorbent integrity for comparative analysis.

### 2.7. Molecular Dynamics (MD) Simulations

Molecular dynamics (MD) simulations were performed to explore the confined diffusion behavior of gas molecules within UiO-type MOFs. First, the Vienna ab initio Simulation Package (VASP 6.4.2) [46] was utilized for the geometric optimization of the Cambridge Crystallographic Data files of U_1_ and U_F4_. The Perdew–Burke–Ernzerhof (PBE) density function [47] in combination with D3 dispersion correction [48] was used in this optimization procedure. The density cut-off was set at 500 Ry, and the overall energy converged to within 10^−6^ eV. To calculate the atomic charges of the frameworks, the Density Derived Electrostatic and Chemical (DDEC6) [49,50] method was adopted. Subsequently, Grand Canonical Monte Carlo (GCMC) simulations were performed by the RASPA code [51] using the polarizable force field parameters based on DREDING and Universal Force Field (UFF) [52]. In the GCMC simulation, both the frameworks and guest molecules were treated as rigid molecules. Specifically, H_2_ and N_2_ molecules were modeled as three-site rigid molecules. All atomic or metal ionic polarizabilities were obtained from references [53,54]. To ensure the accuracy of the force field, global scale factor ζ (ζ = 0.09) and scaling factors λ were induced to adjust the polarizability and the Lennard-Jones energy parameters respectively. This adjustment was crucial to ensure that the experimental isothermal adsorptive data were in good agreement with the simulated results. All MD simulations were performed within the NVT ensemble and were run for a total duration of 1 ns with a time step of 1 fs. The simulations consisted of 1 × 10^6^ cycles, including 2000 initialization cycles and 2000 equilibration cycles.

## 3. Results

### 3.1. Structural Characterization of Isostructural UiO-66 Samples

**Figure 2:**
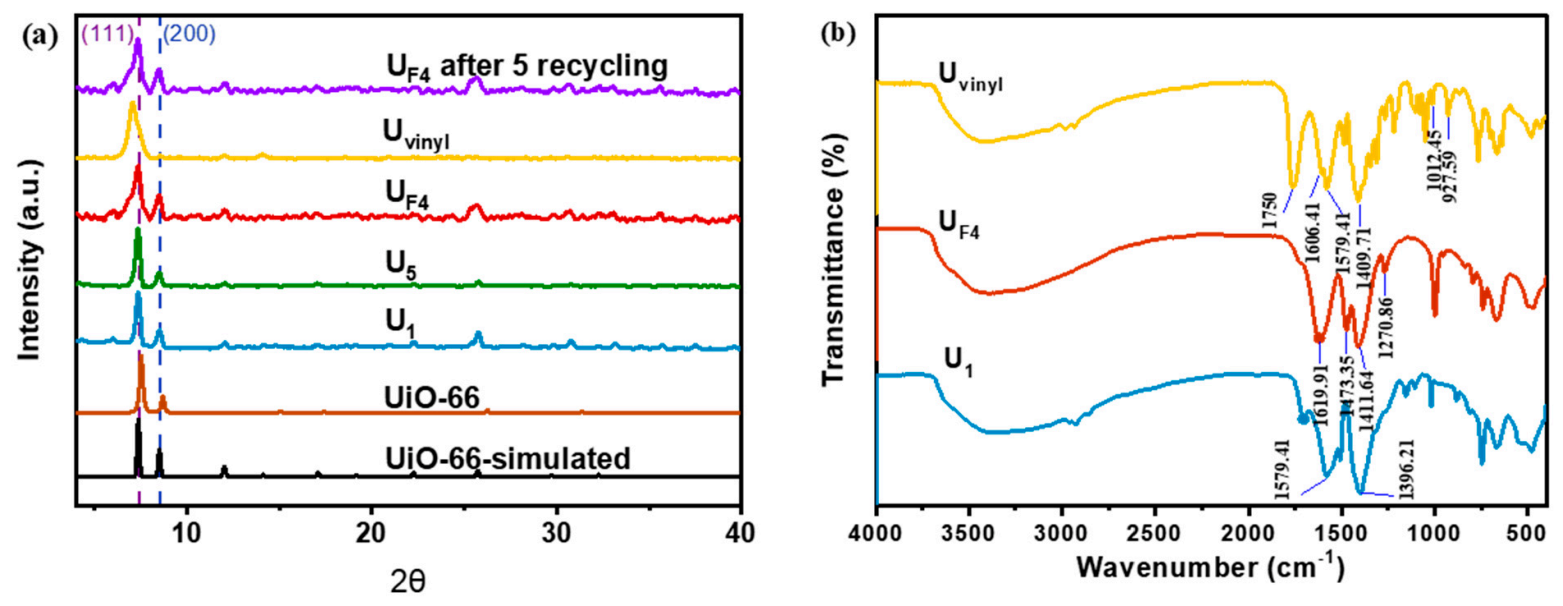
(**a**) PXRD patterns and (**b**) FT-IR spectra of the three isostructural UiO-66 type MOFs.

The crystalline integrity of the synthesized isostructural UiO-66 samples was first evaluated via powder X-ray diffraction (PXRD) analysis (Figure 2a). The observed diffraction peaks matched well with literature-reported values for isostructural UiO-66 frameworks [12,31,37], confirming the successful fabrication of high-purity crystalline materials via the solvothermal synthesis route. The main diffraction peaks are located at 2θ = 7.3° and 8.5°, corresponding to the (111) and (200) planes, respectively. Notably, the absence of extraneous peaks further attests to the absence of secondary phases, underscoring the structural homogeneity of the samples.

Complementary Fourier-transform infrared spectroscopy (FT-IR) analysis (Figure 2b) provided molecular-level insights into the framework composition. UiO-66-type materials show complete disappearance of the broad O-H stretch (2500–3300 cm^−1^), replaced by asymmetric/symmetric COO^−^ vibrations at 1579 cm^−1^ and 1396 cm^−1^, confirming Zr^4+^-carboxylate coordination. All spectra lack free carboxylic acid peaks, confirming deprotonation and framework formation. U_vinyl_ retains vinyl C=C stretch at 1606 cm^−1^ and =CH_2_ deformation at 1012 cm^−1^ and 928 cm^−1^, while its COO^−^ bands shift to 1579 cm^−1^ (ν_as_) and 1410 cm^−1^ (ν_s_), indicating minimal electronic perturbation from conjugation. The adsorption band at 1750 cm^−1^ is also observed in the spectrum of U_vinyl_, which we assign to either a combination band of δ(H_2_O) from trace adsorbed water with framework vibrations, or the C=O stretching vibration of dimers from uncoordinated -COOH groups [55,56]. U_F4_ exhibits a high-frequency shifted ν_as_(COO^−^) at 1620 cm^−1^ (due to F-induced electron withdrawal) and intense C-F stretches (1271 cm^−1^), suggesting a slight alteration in the coordination mode of the carboxylate group with Zr. The distinct splitting of the aromatic C=C skeletal vibration observed at 1473 cm^−1^ and 1412 cm^−1^ can be attributed to the strong electron-withdrawing inductive effect induced by the tetrafluoro substitution pattern. This substitution-induced electronic perturbation disrupts the symmetry of the benzene ring, resulting in vibrational mode separation that manifests as two resolved absorption peaks.

As summarized in Table 1 and Figures S1–S4, the isostructural UiO-66-type samples exhibit gradient characteristics in pore structures. U_1_ (SSA = 1527 m^2^/g, Vp = 0.63 cm^3^/g) and U_5_ (SSA = 1303 m^2^/g, Vp = 0.55 cm^3^/g) exhibited microporous-dominated structures (MPW = 5.1~5.4 Å), with their ultra-high surface area and pore volume making them ideal candidates for gas storage. U_vinyl_ (SSA = 801 m^2^/g, APW = 43.7 Å) showed a comparable average pore width to U_1_ (APW = 42.6 Å) but with significantly enhanced mesoporous contribution. Despite reduced specific surface area (702 m^2^/g) and pore volume (0.41 cm^3^/g), U_F4_ (APW = 79.2 Å) exhibits a shift toward larger pore size distribution. This structural transition is corroborated by the characteristic H_4_-type hysteresis loop in N_2_ adsorption–desorption isotherms (Figure S4), indicative of mesoporous structure rearrangement. U_F4_ shows a much larger BJH average pore width than U_1_, indicating the presence of significant mesoporosity. This mesoporosity mainly comes from ligand defect-induced pore structures. The isostructural UiO-66 materials displayed continuous evolution from microporous to micro-mesoporous structures, with U_1_ achieving an optimal balance between adsorption capacity and mass transfer efficiency, while U_F4_’s structural features may be suitable to address specific applications after weighing relevant considerations.

### 3.2. Adsorption Isotherms

**Figure 3:**
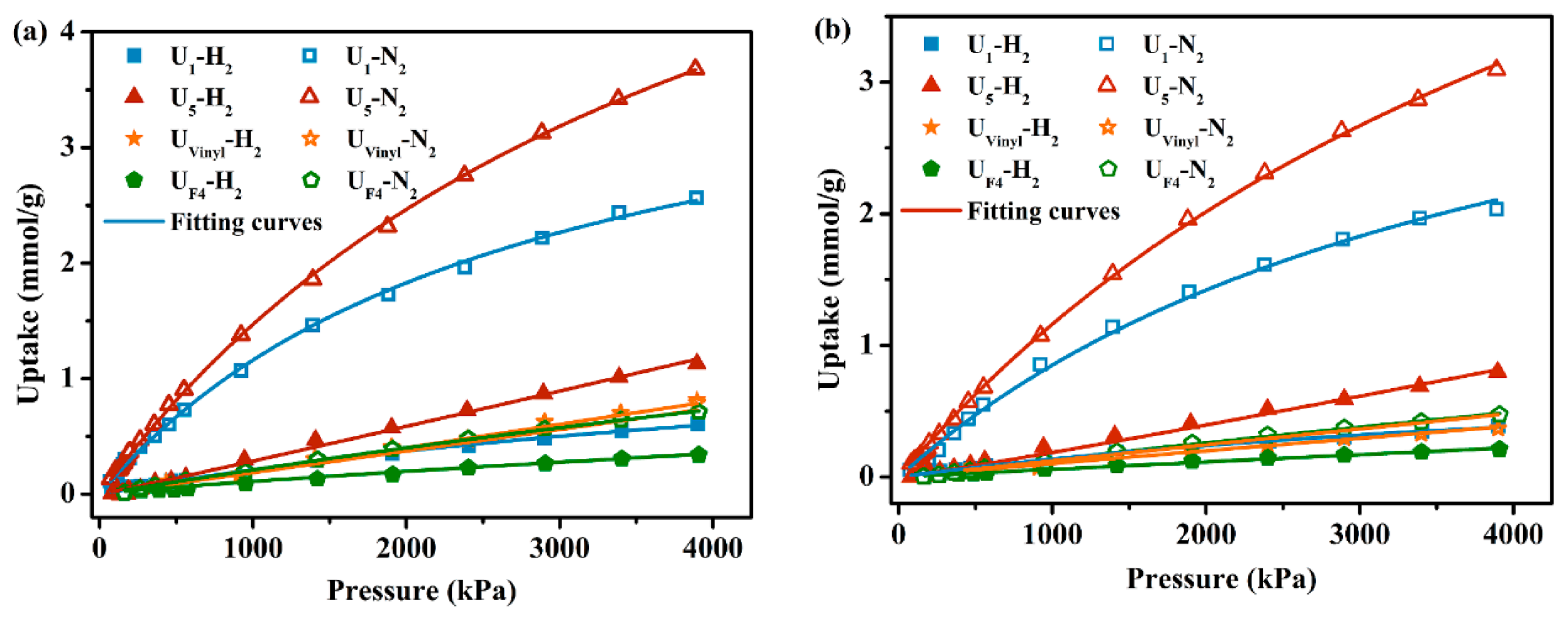
Isothermal raw adsorption data and fitting curves of N_2_ and H_2_ adsorption on U_1_ (blue), U_5_ (red), U_vinyl_ (yellow), and U_F4_ (green) at (**a**) 278 K and (**b**) 298 K (filled symbols: H_2_; open symbols: N_2_).

Adsorption isotherms are crucial for understanding the interaction between adsorbates and adsorbents. To this end, the gas isothermal equilibrium adsorption data of pure N_2_ and H_2_ were measured at 278 K and 298 K, and the results are plotted in Figure 3. Across all experimental conditions, the adsorption capacities of N_2_ were consistently higher than those of H_2_ within the 0–4000 kPa pressure range. It can be attributed to the higher induced adsorption potential formed between zirconium metal and N_2_ molecules via back-bonding interaction [29,57]. Additionally, molecular quadrupole moments and molecular sizes also play significant roles [11]. Both N_2_ and H_2_ uptake showed a downward trend with increasing temperature, indicating that these adsorption processes are typical thermodynamically controlled physisorption. Over the tested pressure and temperature ranges, the order of N_2_ uptake from high to low was: U_5_, U_1_, U_vinyl,_ and U_F4_. For hydrogen, U_5_ exhibited the highest adsorptive capacity, followed by U_vinyl_, U_1,_ and U_F4_. At 278 K and approximately 4000 kPa (Figure 3a), the experimental adsorption capacities of U_5_, U_1_, U_vinyl_ and U_F4_ reached 3.68 (N_2_) and 1.13 (H_2_), 2.56 (N_2_) and 0.61 (H_2_), 0.79 (N_2_) and 0.81 (H_2_), 0.71 (N_2_), and 0.34 (H_2_) mmol/g, respectively. As shown in Figure 3b, at 298 K and 4000 kPa, the adsorption capacity of U_5_ for N_2_ and H_2_ decreased to 3.09 and 0.80 mmol/g, respectively. The N_2_ adsorption capacities of U_vinyl_ and U_F4_ were less remarkable compared to U_1_ and U_5_. This may be because U_1_ and U_5_ have larger pore volumes than U_F4_ and U_vinyl_ (Table 1). In fact, at high pressures, pore volume is a key driving force for adsorption [33]. Moreover, the relatively large adsorption capacity of U_vinyl_ for H_2_ is consistent with the conclusion proposed by Yaghi et al., which states that an expanded π-conjugated system will improve the H_2_ adsorption capacity [58]. Nevertheless, the H_2_ adsorption capacity was still lower than that of N_2_. U_F4_ had the lowest uptake and the smallest slope, which makes it easier to be regenerated. In the low-pressure range, the adsorption isotherms were irregular. This was due to the interference of atmospheric gases that entered the cell when it was transferred from the regeneration station to the analysis port, even though the amounts of gases were extremely small [33]. Given these results, it is challenging to intuitively determine whether the combination of these properties is favorable for the adsorptive separation of N_2_-H_2_ mixture using UiO MOFs. The potential performance of these Zr-based analogs in separation requires further investigation through a combination of theoretical analysis and dynamic breakthrough experiments.

By analyzing the original isothermal adsorption data, the ideal adsorption selectivity of each adsorbent for the N_2_-H_2_ mixture can be assessed based on a specific adsorption model. As depicted in Figure 3 and Figures S13–S16, within the measurement range, the Langmuir model was employed to fit all the experimental isotherms of N_2_, whereas the Freundlich model was used for those of H_2_. The fitted isotherms showed excellent agreement with experimental data (R^2^ ≥ 0.99), and the detailed fitting parameters are presented in Table S1.

### 3.3. Isosteric Heat of Adsorption (Qst) and Ideal Selectivity

**Figure 4:**
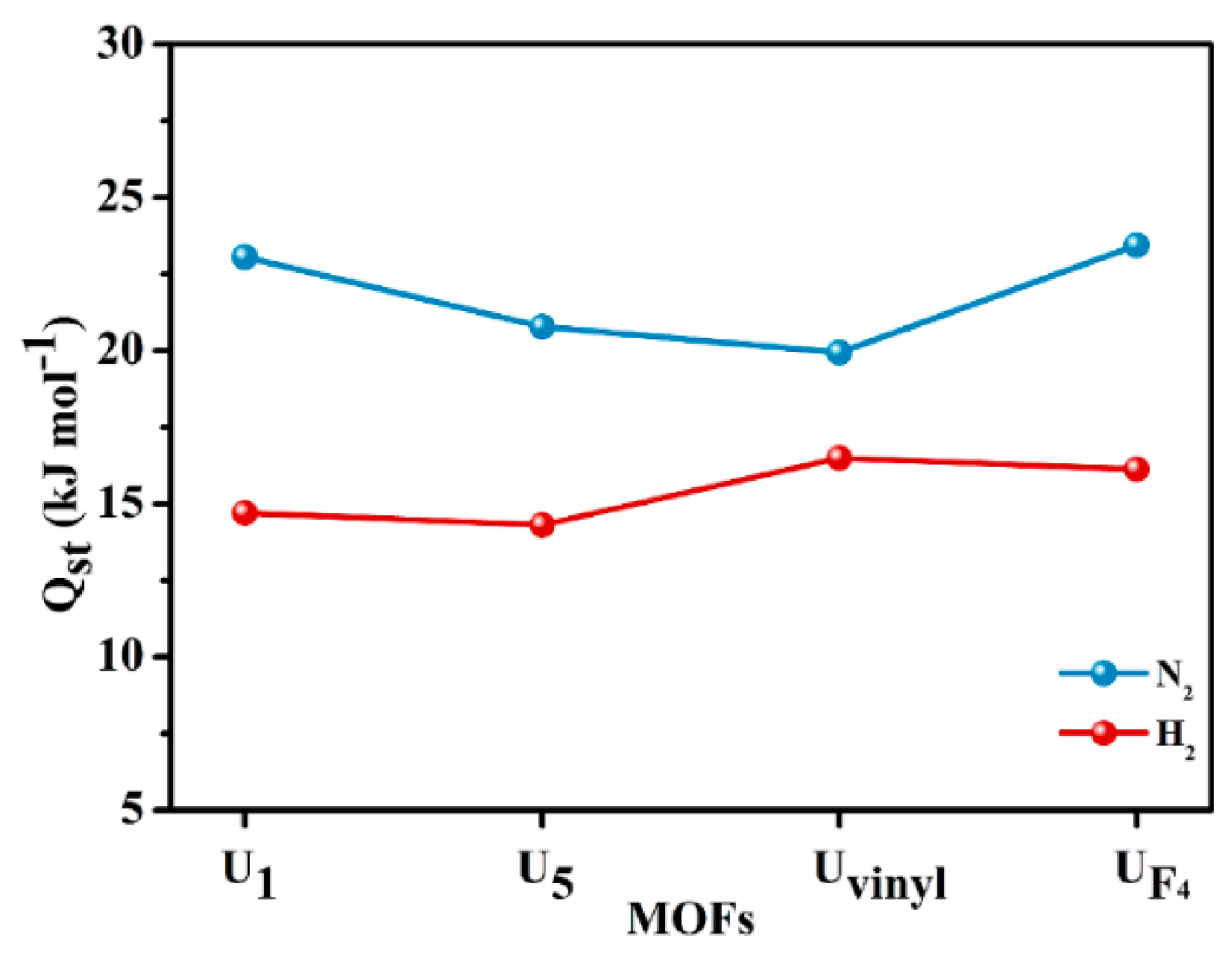
Initial adsorption enthalpies of N_2_ and H_2_ for UiO MOFs at 278 K and 298 K.

Apart from adsorption capacity, the isosteric heat of adsorption (Q*_st_*) is another crucial parameter that helps to understand the trend, state, and spontaneity of the adsorption process, and to evaluate the interaction strength between adsorbate and adsorbent. The relevant parameters for fitting the adsorption heat using the Virial expansion method are detailed in the attached Figures S5–S12. The differences in Q*_st_* for UiO MOFs are shown in Figure 4 and compared at nearly zero coverage (with a loading of 0.002 mmol/g).

As shown in Figure 4, on the given UiO MOFs at a loading of 0.002 mmol/g, the Q*_st_* of N_2_ adsorption was consistently higher than that of H_2_. Among these frameworks, N_2_ binds most strongly to U_F4_, followed by U_1_, U_5,_ and U_vinyl_, with initial adsorption enthalpies of 23.4, 23.0, 20.8, and 19.9 kJ/mol, respectively. For H_2_, the Q*_st_* values in descending order are: U_vinyl_ (16.5 kJ/mol), U_F4_ (16.1 kJ/mol), U_1_ (14.7 kJ/mol), and U_5_ (14.3 kJ/mol), indicating that the preferential adsorption of H_2_ molecules by U_vinyl_ and U_F4_ was more significant. U_vinyl_ and U_5_, especially U_vinyl_, showed relatively small differences in the initial adsorption heat of H_2_ and N_2_, suggesting weak differential adsorption effects. U_1_ and U_F4_, by contrast, exhibited stronger differential adsorption effects. Overall, the combined effects of pore size changes and the introduction of functional groups on the initial adsorption enthalpies may outweigh the negative effects of increased coordination defects on the adsorption heat. Naturally, the main interaction between the material and adsorbate is Coulomb attraction, resulting from the synergy of open metal sites (OMS), functionalized pores, and controlled preparation [28].

**Figure 5:**
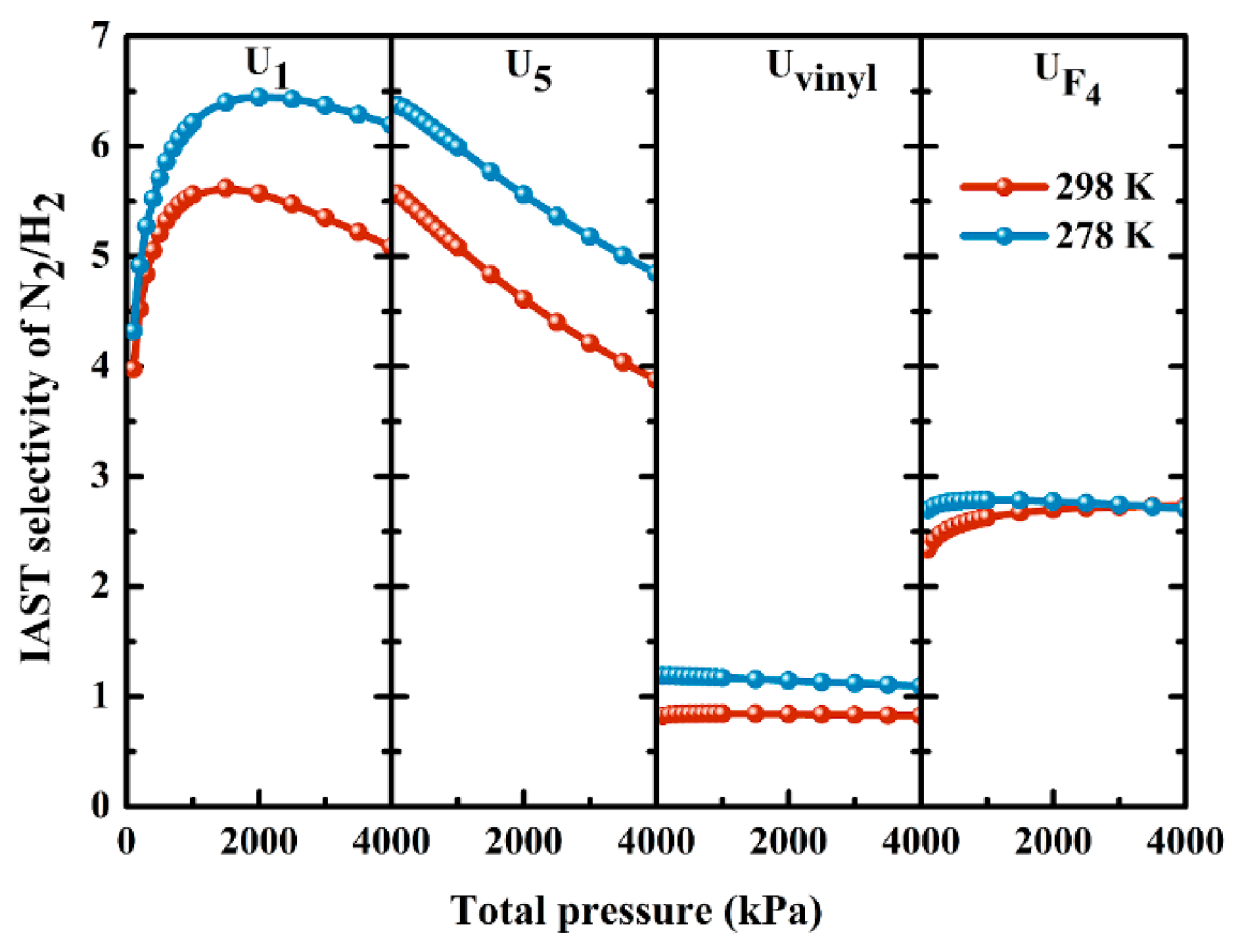
IAST predicted selectivity of N_2_/H_2_ (2/1, *v*/*v*) adsorption on Zr-based MOFs at 278 K and 298 K.

Figure 5 exhibits the IAST selectivity of N_2_-H_2_ mixture (N_2_/H_2_, 2/1, *v*/*v*) on Zr-based MOFs at 278 K and 298 K. As depicted, the N_2_/H_2_ selectivity values of the UiO MOFs at 278 K were higher than those at 298 K. U_1_ and U_5_ had the highest S_N2/H2_ values, reaching about 6.5 at 278 K, and remaining above 4.0 even when the pressure approached 4000 kPa. Notably, the S_N2/H2_ values of U_1_ increased with pressure, peaking at 6.4 at 2000 kPa and 298 K. This may be related to nonnegligible adsorbate–adsorbate interactions at low coverage [45]. The experimental adsorption selectivity value was close to the simulated value (about 8.0 at 20 bar and 298 K, N_2_/H_2_, 3/7, *v*/*v*) in reference [7]. However, from an economic perspective of the adsorption process, selectivities greater than 4.0 have limited practical significance [17]. For U_F4_, the N_2_/H_2_ separation selectivity was about 3 across the entire 0~4000 kPa pressure range, with higher selectivity at low pressures. Compared with other Zr-based MOFs, U_vinyl_ exhibited a low predicted selectivity of about 1.0, which may be attributed to the fact that the enlarged π-conjugated system increases the H_2_ adsorbed amount (*n*H_2_), and this was consistent with the adsorption heat and *n*N_2_/*n*H_2_. For a separation factor less than 2.0, it is challenging to design a satisfactory adsorption separation process [17]. Notably, since the adsorption amount of N_2_ and H_2_ on Zr-based MOFs is low at low pressure, especially U_F4_ and U_vinyl_, there are some uncertainties in the single-component adsorption isotherm fitting.

### 3.4. Dynamic Column Breakthrough Experiments

**Figure 6:**
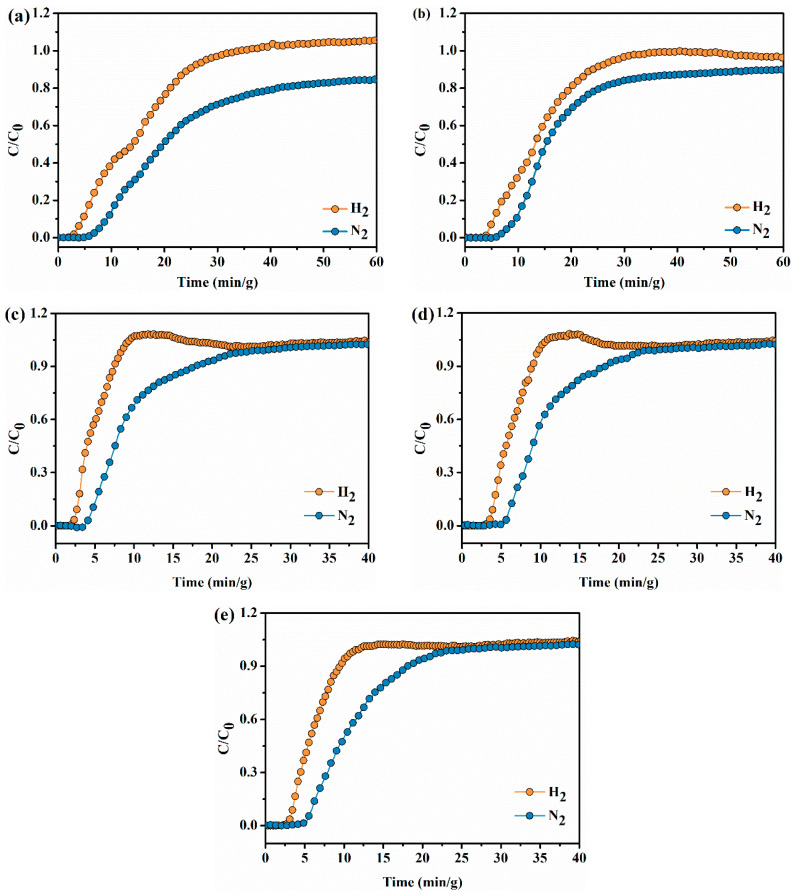
Breakthrough curves of U_1_ at 298 K for the mixtures of (**a**) N_2_/H_2_/He (10/10/80, vol%, the same below; Flow rate: 30 mL/min) at 5 bar; (**b**) N_2_/H_2_/He (20/10/70; Flow rate: 30 mL/min) at 5 bar; (**c**) N_2_/H_2_/He (10/20/70; Flow rate: 25 mL/min) at 5 bar; (**d**) N_2_/H_2_/He (10/20/70; Flow rate: 25 mL/min) at 10 bar and (**e**) N_2_/H_2_/He (10/20/70; Flow rate: 25 mL/min) at 15 bar.

**Figure 7:**
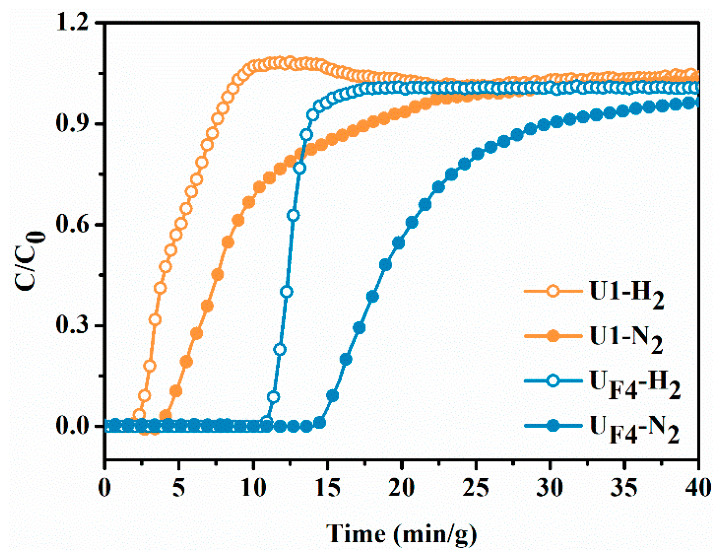
Breakthrough curves for N_2_/H_2_/He (10/20/70, vol%; Flow rate: 25 mL/min) mixture in U_1_ and U_F4_ at 298 K and 5 bar.

Based on the preceding discussion, U_1_ and U_F4_ were selected for multicomponent dynamic column breakthrough measurements to evaluate their N_2_/H_2_ separation performance at 298 K and 5 bar. As shown in Figure 6, U_1_ exhibited limited N_2_/H_2_ separation capability across all tested gas mixtures (N_2_/H_2_/He ratios: 10/10/80, 20/10/70, 10/20/70, vol%). The breakthrough profiles revealed that N_2_ displacement occurred before H_2_ reached adsorption equilibrium, with gas concentration variations only marginally affecting the retention time difference (Δτ = 1.1–2.5 min/g). Increasing H_2_ concentration to 20% induced a roll-up effect in its breakthrough curves (Figure 6c), suggesting weak H_2_-framework interactions and low H_2_ adsorption capacity. While the distinctive hump in Figure 6c,d hinted at potential kinetic separation under 10/20/70 vol% conditions [59,60], pressure elevation to 15 bar eliminated this feature (Figure 6e), demonstrating the impracticality of dynamic separation at elevated pressures. The distended breakthrough curves (Figure 6) indicated intra-crystalline diffusion limitations [61], confirming incomplete thermodynamic equilibrium in the fixed-bed system. In contrast, U_F4_ demonstrated superior separation performance under identical conditions (N_2_/H_2_/He, 10/20/70, vol%; 25 mL/min, 298 K, 5 bar). As depicted in Figure 7, U_F4_ achieved clear N_2_/H_2_ separation with N_2_ retention time reaching 3.5 min/g, enabling pure H_2_ recovery during the initial elution phase. The ratio of breakthrough time differential to operating pressure (Δτ/ΔP = 0.7 min·g^−1^·bar^−1^) exceeds the PSA process threshold of 0.5, confirming superior pressure-responsive behavior for industrial-scale gas separation. This performance difference arises not only from the affinity between the adsorbent and adsorbate but also from differential gas molecule diffusion rates within the U_1_ and U_F4_ frameworks.

### 3.5. MD Simulations

**Figure 8:**
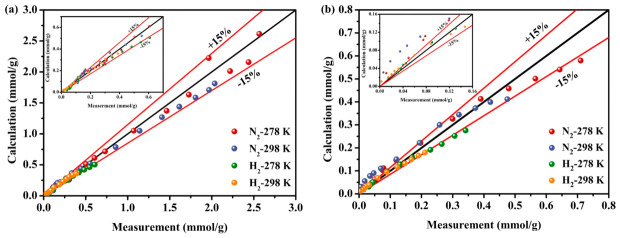
Comparison of the simulated and measured values of isothermal adsorption on (**a**) U_1_ and (**b**) U_F4_.

In GCMC simulations, the N_2_-to-H_2_ molar ratio in both U_1_ and U_F4_ materials is 1:2, consistent with the dynamic breakthrough experiments. The polarizability was calibrated using a global scale factor (ζ = 0.09), while Lennard-Jones energy parameters were scaled by λ to minimize experimental-simulation discrepancies. All polarizable force field parameters and atomic/ionic polarizabilities are provided in Table S2. Figure 8 compares simulated and experimental isothermal adsorption data, showing deviations within ±15% across most pressure conditions.

**Figure 9:**
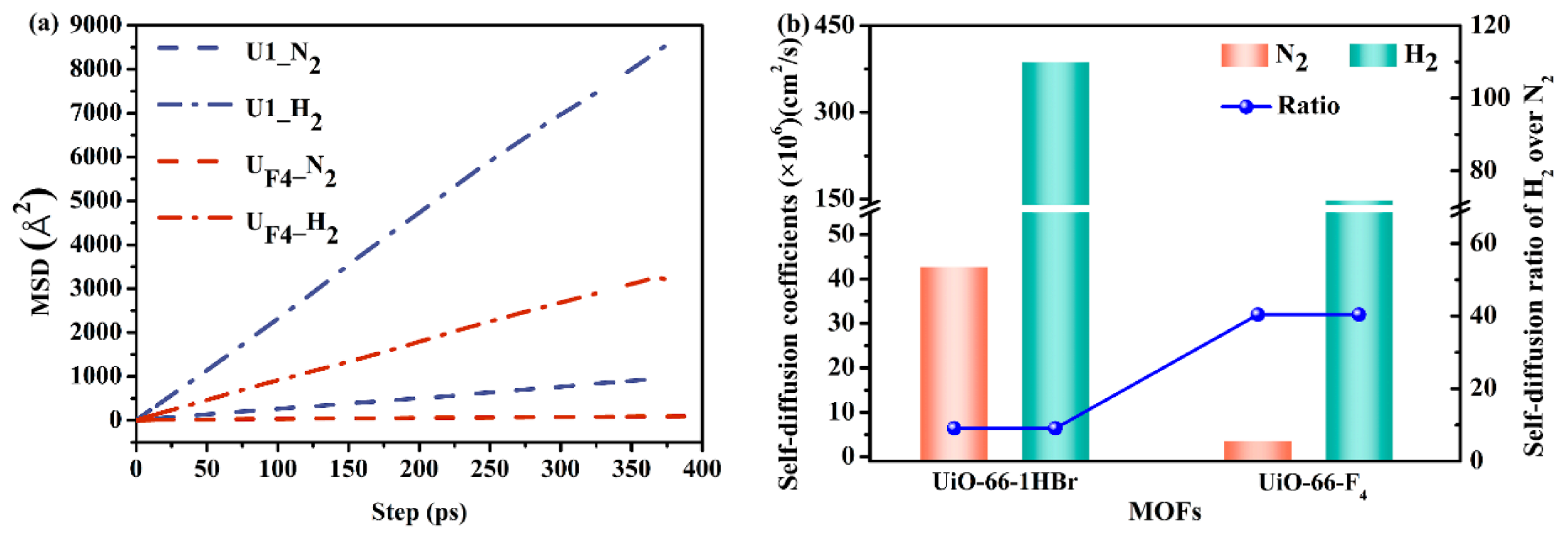
(**a**) Mean-square displacements for N_2_-H_2_ mixture (N_2_/H_2_, 10/20, *v*/*v*) and (**b**) self-diffusion coefficients of H_2_-N_2_ and its ratio in U_1_ and U_F4_ at 298 K.

MD simulations reveal distinct gas diffusion behaviors within the adsorbents (Figure 9). The relatively fast diffusion of H_2_ in U_1_ is attributed to its high surface area and pore volume (Table 1), which minimizes steric hindrance, and its weak H_2_-framework interaction (Q_st_ = 14.7 kJ/mol, Figure 4), which reduces surface residence time. The calculated self-diffusion coefficients for H_2_ and N_2_ in material U_1_ were 3.87 × 10^−4^ cm^2^/s and 4.27 × 10^−5^ cm^2^/s, respectively, yielding a diffusion selectivity (D_H2_/D_N2_) of 9.05. In contrast, material U_F4_ exhibited significantly lower diffusivities of 1.48 × 10^−4^ cm^2^/s for H_2_ and 3.66 × 10^−6^ cm^2^/s for N_2_, resulting in a markedly higher diffusion selectivity of 40.38. This superior H_2_ diffusivity drives its preferential occupation of pore space during the initial adsorption phase, which aligns with competitive breakthrough experiments demonstrating an H_2_ elution time advantage (Δτ = 3.5 min·g^−1^). The pronounced difference in gas diffusivity, particularly within U_F4_, underscores its kinetic preference for H_2_ during adsorption initiation. This simulation result also shows good agreement with the dynamic penetration experiment.

However, thermodynamic equilibrium favors N_2_ adsorption due to its stronger interactions with the framework, as evidenced by the difference in isosteric heats of adsorption (ΔQ_st_ (N_2_-H_2_): 8.3 kJ·mol^−1^, U_1_; 5.3 kJ·mol^−1^, U_F4_). This contrast highlights U_F4_’s superior kinetic selectivity towards H_2_ over N_2_ compared to U_1_. Crucially, although both adsorbents exhibit higher thermodynamic selectivity for N_2_, the MD results confirm that the N_2_-H_2_ separation mechanism in these micro/mesoporous Zr-MOFs is primarily kinetically controlled.

The efficient N_2_/H_2_ separation achieved by U_F4_ stems from a synergistic mechanism: strong micropore confinement effectively captures N_2_ (reflected in a van der Waals interaction energy), while the mesoporous structure and the fluorinated pore facilitate accelerated H_2_ transport. This combination enables U_F4_ to meet the kinetic performance requirements (Δτ > 3 min·g^−1^) for industrial PSA processes. Furthermore, U_F4_’s enhanced N_2_/H_2_ adsorption selectivity, validated by competitive dynamic adsorption experiments, establishes it as a viable adsorbent for practical N_2_/H_2_ separation applications.

### 3.6. Cyclic Adsorption Performance Assessment

For practical separation applications, adsorbents must exhibit robust regeneration capability and structural stability. To evaluate the cyclic performance of U_F4_, breakthrough experiments employing a N_2_/H_2_/He gas mixture (10/20/70, vol%; 25 mL/min) were conducted for 5 consecutive cycles at 298 K and 5 bar. Between each breakthrough run, the adsorption column was regenerated under 10 mL/min of He gas flow at 453 K (setpoint of the external heater, estimated bed temperature ~393 K) for 3 h.

**Figure 10:**
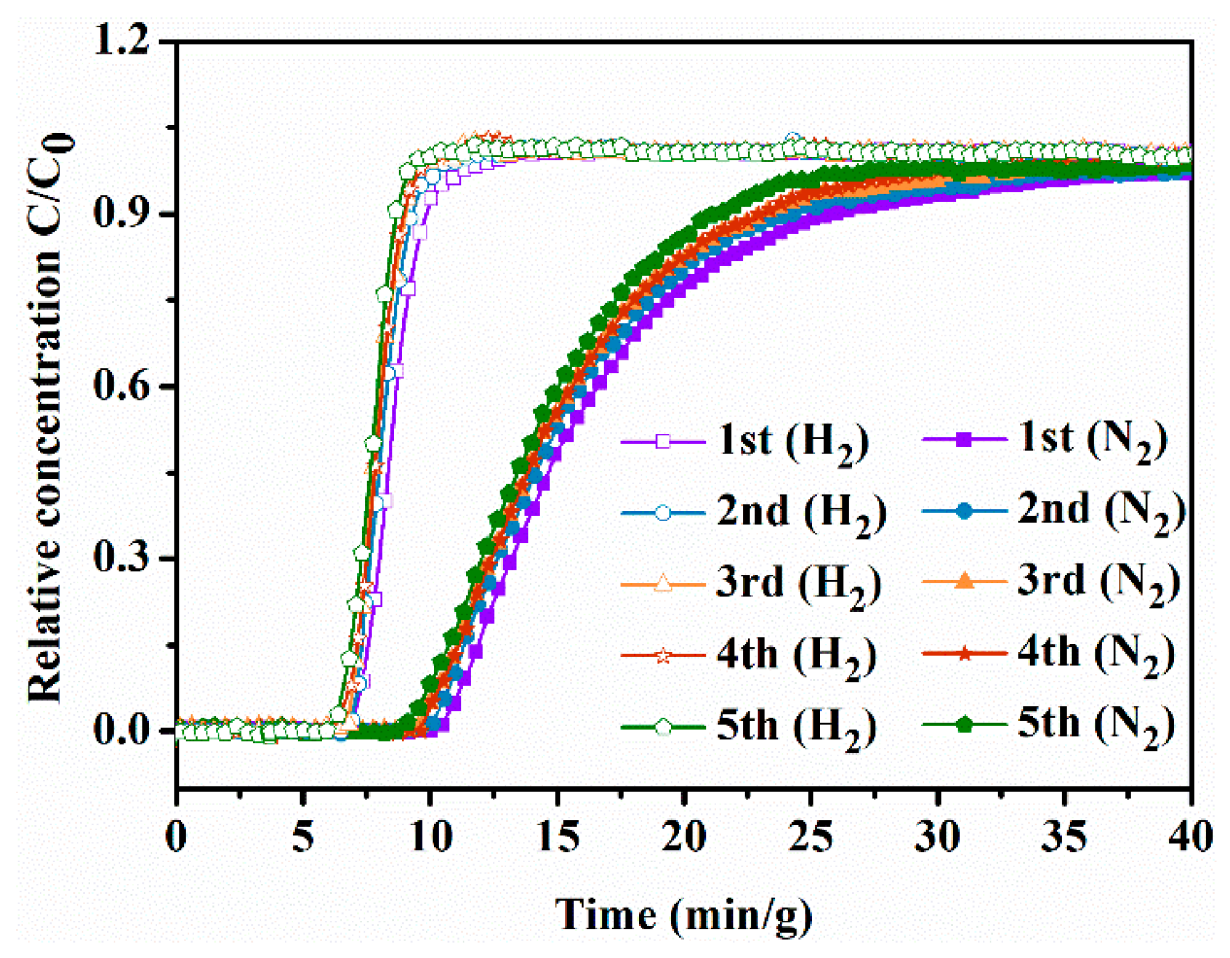
Breakthrough circulation test results for N_2_/H_2_/He (10/20/70, vol%) mixture on an adsorption column of U_F4_ at 298 K and 5 bar.

As illustrated in Figure 10, the breakthrough curves and the characteristic breakthrough time for both N_2_ and H_2_ showed negligible variation across the 5 cycles. Furthermore, as confirmed by the PXRD spectra (Figure 2), the framework structure and characteristic morphology of U_F4_ remained intact after the cyclic testing. This exceptional stability and reproducible performance under repeated adsorption-regeneration conditions strongly supports the practical viability of U_F4_ for gas separation applications.

## 4. Conclusions

The present study demonstrates the potential application of UiO-66-F_4_ (U_F4_) for the selective separation of N_2_-H_2_ gas mixtures. This material exhibits excellent stability and strong adsorbate binding affinity. To evaluate its practical performance, breakthrough experiments were combined with molecular dynamics (MD) simulations. The breakthrough results reveal that U_F4_ possesses higher selectivity towards N_2_ over H_2_, enabling the production of high-purity hydrogen gas from N_2_-H_2_ mixtures under specific conditions. The MD simulation results corroborated the breakthrough experimental findings for N_2_/H_2_ separation. Furthermore, this work highlights the promising prospects of breakthrough separation technology for addressing the N_2_/H_2_ separation challenge. However, research on N_2_/H_2_ separation using MOFs as adsorbents, particularly under high-flux conditions and employing breakthrough experiments, remains limited, and significant work is still required.

## Figures and Tables

**Table 1:** Adsorbents properties: BET specific surface area (SSA), pore volume (V_p_), HK Median pore width (MPW), and BJH desorption average pore width (APW).

Adsorbent	Sample Designation	SSA (m^2^/g)	V_p_ (cm^3^/g)	MPW (Å)	APW (Å)
UiO-66-1HBr	U_1_	1527	0.63	5.1	42.6
UiO-66-5HBr	U_5_	1303	0.55	5.4	38.2
UiO-66-CH=CH_2_	U_vinyl_	801	0.45	4.8	43.7
UiO-66-F_4_	U_F4_	702	0.41	4.2	79.2

## Data Availability

The original contributions presented in this study are included in the article/Supplementary Material. Further inquiries can be directed to the corresponding authors.

## Supplementary Materials

The following supporting information can be downloaded at: https://www.mdpi.com/article/10.3390/ma19112418/s1.
