# Supplementary material for: Utilization of Robust Zr-Based Metal–Organic Framework for Efficient N2/H2 Separation
Source: Materials (Basel). 2026 Jun 5;19(11):2418. doi: 10.3390/ma19112418 (PMC13258371; doi:10.3390/ma19112418)
Supplement: Supplementary file 1 [file materials-19-02418-s001.zip › materials-4320851-supplementary.pdf]

Supporting Information

Utilization of robust Zr-based metal-organic framework for efficient N_2/H_2 separation

Xia Chen, Zhilu Wang, Tianhao Wang, Wenxin Ma, Qiang Fu*, Bao-Ju Wang*

School of Chemistry and Chemical Engineering, Shandong University of Technology, Zibo 255000, PR China

* Corresponding author. Tel: + 17611104084

E-mail address: baojuwang@sdut.edu.cn; fuqiang@tju.edu.cn.

Table of Contents

Table S1. Estimated parameter values for the fitting of pure N_2 and H_2 isothermal adsorption data on Zr-based MOFs by the Langmuir (N_2) and Freundlich (H_2) model.

Table S2. Generic force field parameters for H_2, N_2 and other atom types corresponding to those in U_1 and U_{F4}.

Fig. S1. (a) N_2 adsorption isotherm of activated U_1 at 77 K; pore size distribution calculated from (b) HK model and (c) BJH desorption curve.

Fig. S2. (a) N_2 adsorption isotherm of activated U_5 at 77 K; pore size distribution calculated from (b) HK model and (c) BJH desorption curve.

Fig. S3. (a) N_2 adsorption isotherm of activated U_{vinyl} at 77 K; pore size distribution calculated from (b) HK model and (c) BJH desorption curve.

Fig. S4. (a) N_2 adsorption isotherm of activated U_{F_4} at 77 K; pore size distribution calculated from (b) HK model and (c) BJH desorption curve.

Fig. S5. Fitted N_2 adsorption isotherms of U_1 measured at 278 K and 298 K.

Fig. S6. Fitted H_2 adsorption isotherms of U_1 measured at 278 K and 298 K.

Fig. S7. Fitted N_2 adsorption isotherms of U_5 measured at 278 K and 298 K.

Fig. S8. Fitted H_2 adsorption isotherms of U_5 measured at 278 K and 298 K.

Fig. S9. Fitted N_2 adsorption isotherms of U_{F_4} measured at 278 K and 298 K.

Fig. S10. Fitted H_2 adsorption isotherms of U_{F_4} measured at 278 K and 298 K.

Fig. S11. Fitted N_2 adsorption isotherms of U_{vinyl} measured at 278 K and 298 K.

Fig. S12. Fitted H_2 adsorption isotherms of U_{vinyl} measured at 278 K and 298 K.

Fig. S13. Langmuir fitting of N_2 isotherms and Freundlich fitting of H_2 isotherms for U_1.

Fig. S14. Langmuir fitting of N_2 isotherms and Freundlich fitting of H_2 isotherms for U_5.

Fig. S15. Langmuir fitting of N_2 isotherms and Freundlich fitting of H_2 isotherms for U_{Vinyl}.

Fig. S16. Langmuir fitting of N_2 isotherms and Freundlich fitting of H_2 isotherms for U_{F4}.

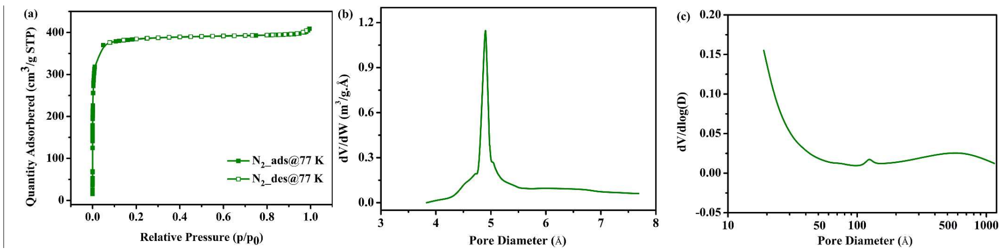

Fig. S1. (a) N_2 adsorption isotherm of activated U_1 at 77 K; pore size distribution calculated from (b) HK model and (c) BJH desorption curve.

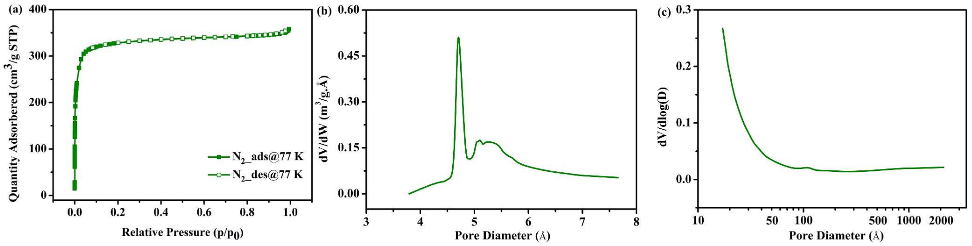

Fig. S2. (a) N_2 adsorption isotherm of activated U_5 at 77 K; pore size distribution calculated from (b) HK model and (c) BJH desorption curve.

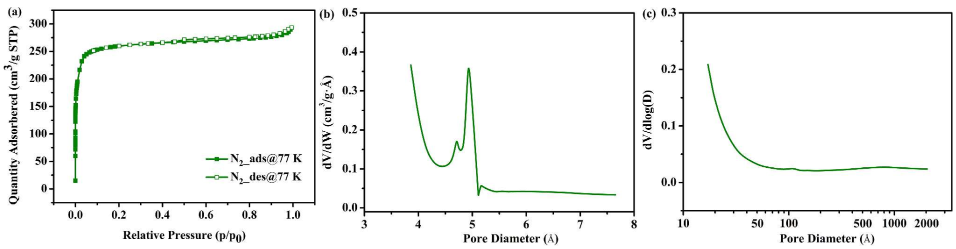

Fig. S3. (a) N_2 adsorption isotherm of activated U_{vinyl} at 77 K; pore size distribution calculated from (b) HK model and (c) BJH desorption curve.

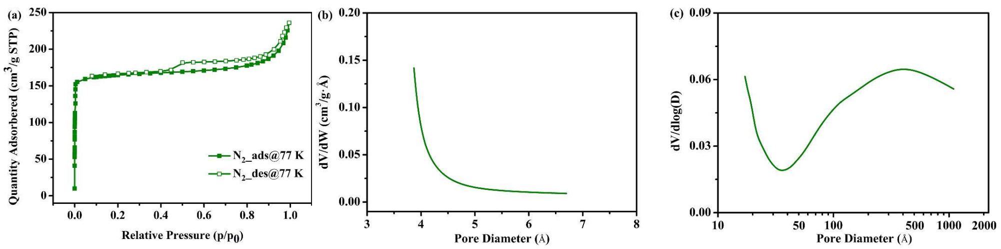

Fig. S4. (a) N_2 adsorption isotherm of activated U_{F_4} at 77 K; (b) HK model and (c) BJH desorption curve.

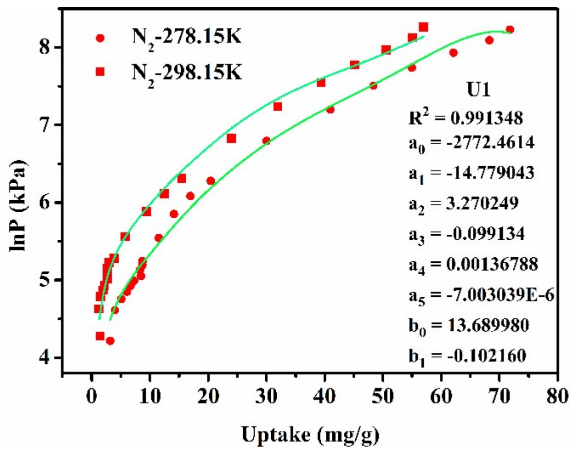

Fig. S5. Fitted N_2 adsorption isotherms of U_1 measured at 278 K and 298 K.

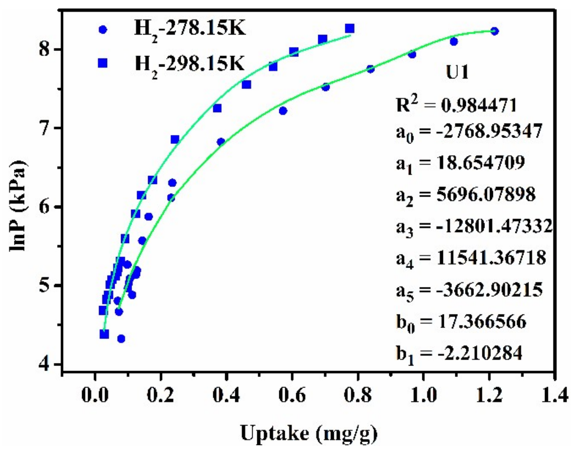

Fig. S6. Fitted H_2 adsorption isotherms of U_1 measured at 278 K and 298 K.

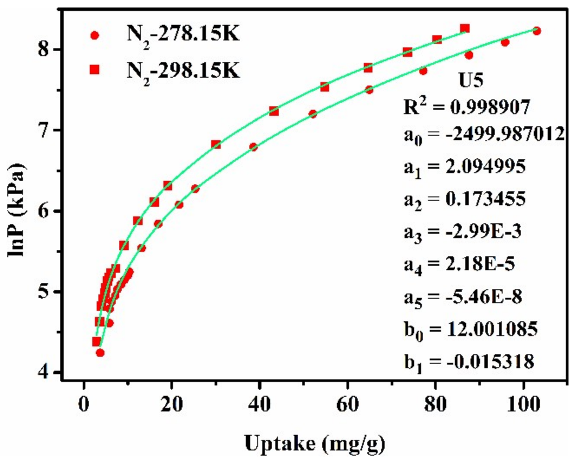

Fig. S7. Fitted N_2 adsorption isotherms of U_5 measured at 278 K and 298 K.

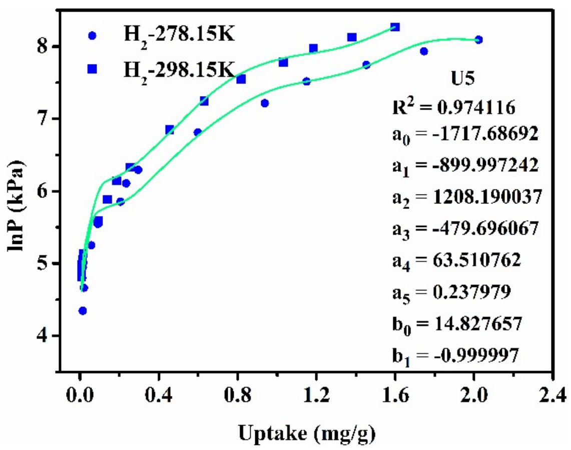

Fig. S8. Fitted H_2 adsorption isotherms of U_5 measured at 278 K and 298 K.

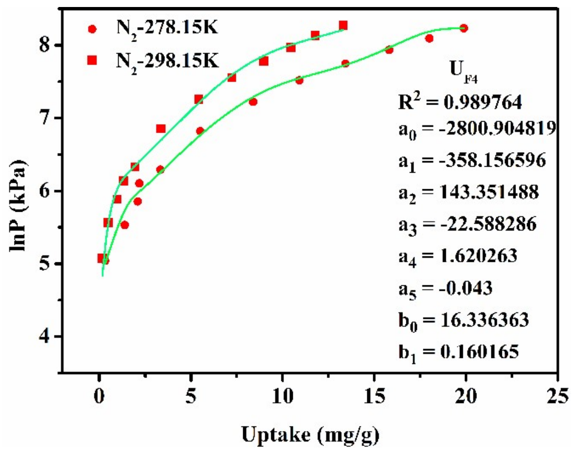

Fig. S9. Fitted N_2 adsorption isotherms of U_{F_4} measured at 278 K and 298 K.

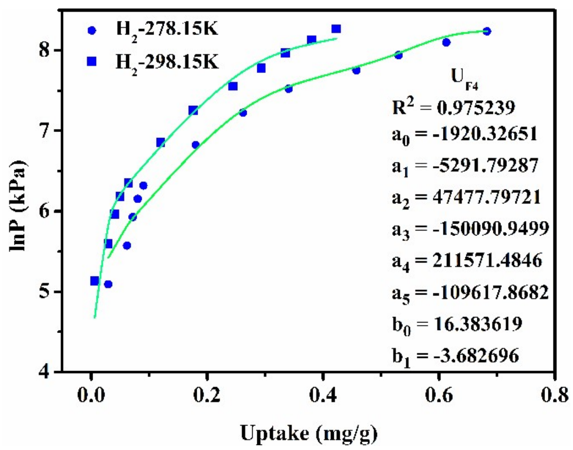

Fig. S10. Fitted H_2 adsorption isotherms of U_{F_4} measured at 278 K and 298 K.

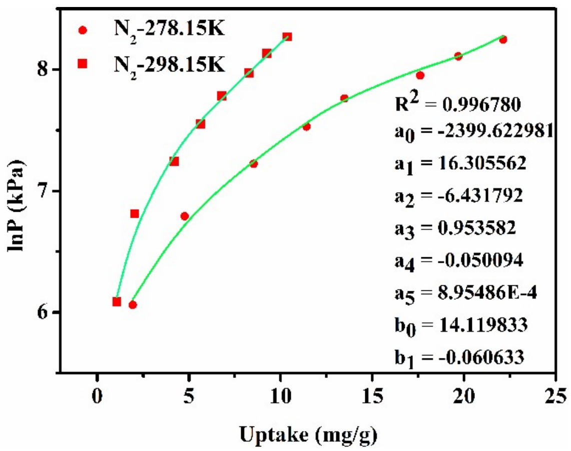

Fig. S11. Fitted N_2 adsorption isotherms of U_{vinyl} measured at 278 K and 298 K.

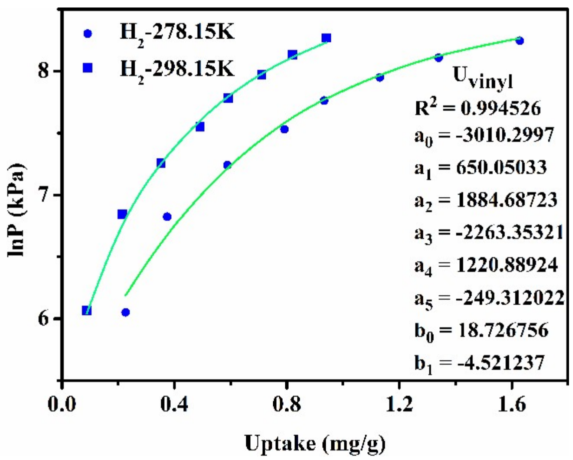

Fig. S12. Fitted H_2 adsorption isotherms of U_{vinyl} measured at 278 K and 298 K.

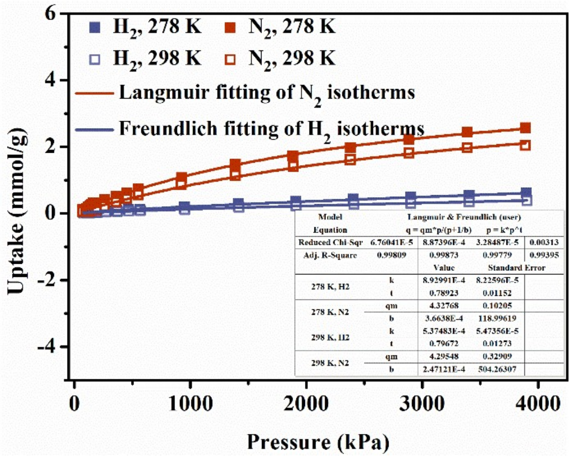

Fig. S13. Langmuir fitting of N_2 isotherms and Freundlich fitting of H_2 isotherms for U_1 at 278 K (filled points) and 298 K (open points).

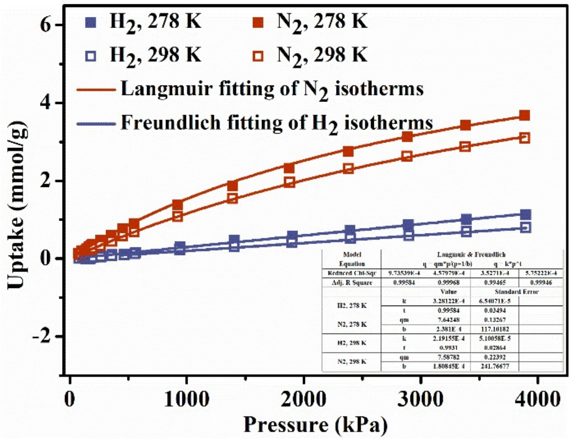

Fig. S14. Langmuir fitting of N_2 isotherms and Freundlich fitting of H_2 isotherms for U_5 at 278 K (filled points) and 298 K (open points).

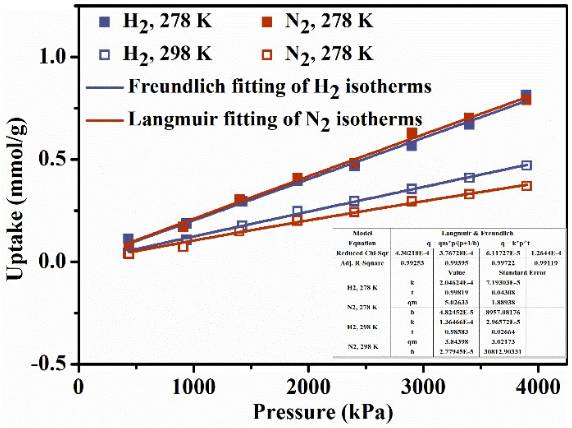

Fig. S15. Langmuir fitting of N_2 isotherms and Freundlich fitting of H_2 isotherms for U_{vinyl} at 278 K (filled points) and 298 K (open points).

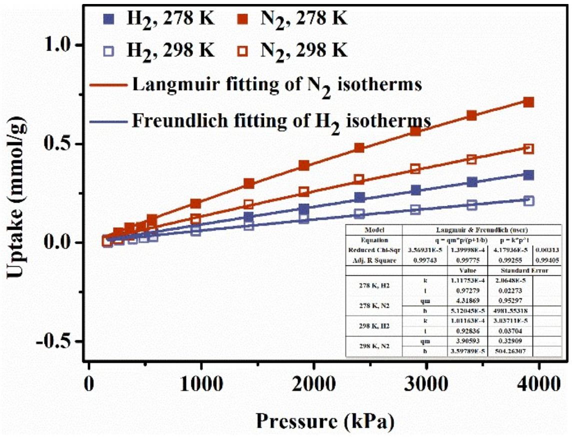

Fig. S16. Langmuir fitting of N_2 isotherms and Freundlich fitting of H_2 isotherms for U_{F_4} at 278 K (filled points) and 298 K (open points).

Table S1. Estimated parameter values for the fitting of pure N_2 and H_2 isothermal adsorption data on Zr-based MOFs by the Langmuir (N_2) and Freundlich (H_2) model.

Adsorbents	Temperature	H_2			N_2		
		k	t	R^2	q_m	b	R^2
U1	278 K	8.92991E-4	0.78923	0.99809	4.32768	3.66380E-4	0.99873
	298 K	5.37483E-4	0.79672	0.99779	4.29548	2.47121E-4	0.99395
U5	278 K	3.28122E-4	0.99584	0.99584	7.64248	2.38100E-4	0.99968
	298 K	2.19155E-4	0.9931	0.99465	7.58782	1.80845E-4	0.99946
U_{vinyl}	278 K	2.04624E-4	0.99819	0.99253	5.02633	4.82452E-5	0.99395
	298 K	1.36466E-4	0.98583	0.99722	3.84398	2.77945E-5	0.99119
U_{F4}	278 K	1.11753E-4	0.97279	0.99743	4.31869	5.12045E-5	0.99775
	298 K	1.01163E-4	0.92836	0.99255	3.90593	3.59789E-5	0.99405

Table S2. Generic force field parameters for H_2, N_2 and other atom types corresponding to those in U_1 and U_{F4}.

Atom type	q(|e|)	δ (Å)	ε (K)
C		47.8562	3.47299
O		48.1581	3.03315
H		7.64893	2.84642
Zr[a]		34.7221	2.78317
F		36.4834	3.0932
H		10.00	2.72
H_com			
N		38.298	3.306
N_com			

a: UFF force field
